# Social anxiety and emoji use: gender differences and the role of loneliness in digital communication among college students

**DOI:** 10.3389/fpsyg.2025.1626509

**Published:** 2025-10-23

**Authors:** Alexis M. Bendl, Ronan M. Cunningham, Lauren Hill, Johanna M. Jarcho

**Affiliations:** ^1^Department of Psychology and Neuroscience, Temple University, Philadelphia, PA, United States; ^2^Department of Psychology and Neuroscience, University of North Carolina, Chapel Hill, NC, United States; ^3^Department of Psychology, Stony Brook University, New York, NY, United States

**Keywords:** digital communication, social anxiety, loneliness, emoji use, computer-mediated communication

## Abstract

Emojis are one of the fastest growing forms of digital communication. However, sending texts can provoke distress. This may be particularly true for the 40% of emerging adults with clinically significant symptoms of social anxiety who often suffer from loneliness and altered communication styles. We hypothesize that to mitigate distress, higher levels of social anxiety and loneliness may be associated with greater emoji use when sending texts that could be interpreted negatively. We also hypothesize the relation would be especially pronounced in females given higher rates of social anxiety and emoji use in general. College students (*N* = 191, 64% female, 18–24 years of age) read a series of vignettes with ambiguous texts and were asked to imagine sending them to a friend. They were instructed to convey positive meaning for half of the texts and negative meaning for the other half. Participants replied with a pre-written response and were given the option to send the response as is or add an emoji expressing a facial expression ranging from happy to displeased. Social anxiety and loneliness were assessed using established self-report scales. Multiple linear regression [*R*^2^ = 0.072, *F*(3,107) = 3.859, *p* = 0.012] demonstrated that women with high levels of both social anxiety and loneliness used emojis more frequently, and that this varied depending on valence. Specifically, women with high social anxiety used emojis more frequently when conveying positively valanced messages (*β* = 0.338, *p* = 0.003). No relations emerged between social anxiety, loneliness and emoji use in men [*R*^2^ = 0.044, *F*(3,52) = 1.844, *p* = 0.151]. Although the effect sizes are small, these findings may inform the design of future studies on mental health and digital communication, increase understanding about gender differences in online communication style, and provide preliminary insights for the development of digital interventions that identify young adults who may most benefit from clinical engagement.

## Introduction

Over the past two decades, computer-mediated communication (CMC) has increased exponentially, with the number of Americans who used at least one social media platform surging from 5% in 2005 to over 70% today (Pew Research Center, n.d.). Use of emojis – symbols of faces, people, or objects used as a digital substitute for non-verbal emotion expression – have also dramatically increased since their 2011 introduction to iOS devices (Hånberg Alonso and Emoji Timeline, 2017). Although some critics claim CMC results in negative psychosocial symptoms, research on the real-world impact of emojis is scarce (Anderson and Rainie, 2018). Additionally, most studies on CMC analyze the emotional impact of receiving digital communication rather than examining the effect of individual differences, like mental health issues, on digital communication style (Kingsbury and Coplan, 2016; Bai et al., 2019). These potential differences in CMC could have implications for those with social anxiety – one of the most common mental disorders in adolescents and young adults – and for those experiencing high levels of loneliness (Leichsenring and Leweke, 2017; Stein and Stein, 2008). The present study examines how social anxiety and loneliness relate to CMC style. We also examined if these relations differed by gender given well established gender-related differences in social anxiety symptoms and communication style (Weinstock, 1999; Asher et al., 2017; Sun et al., 2020). To our knowledge, this is the first to true experiment to quantify the relation between these critical dimensions of mental health and social functioning with emoji usage during text-based conversation. Moreover, it is one of only a few studies to examine emoji use from the perspective of a sender rather than a recipient. These foundational data provide novel insight into the complex interaction between mental and social health and digital communication and may inform future research on how social anxiety and loneliness are expressed through CMC.

## Digital communication

Digital communication and social media have validated benefits, such as increased connectedness within long-distance relationships and facilitation of collective action on important issues (Pettigrew, 2009; Foster, 2015). However, early text-based platforms could not convey non-verbal cues such as body language and facial expression, which are critical components of social communication (Phutela, 2015). As a substitute, pictograms of faces and objects called “emojis” emerged (Hånberg Alonso and Emoji Timeline, 2017; Tauch and Kanjo, 2016; Pardes, 2018). Use of emojis on social media and in text messaging today remains prevalent, with 20% of all tweets in 2021 containing at least one of these characters, suggesting their popularity for the enrichment of short CMC posts and messages (Broni and Emojipedia, 2021). The popularity of emojis may come from their ability to supplement the non-verbal cues that are lost through CMC. Indeed, neuroimaging studies demonstrate that sentences with emojis generate patterns of brain function similar to face-to-face interactions (Yuasa et al., 2011; Gantiva et al., 2020). Some studies even show that emojis can not only match the emotionality and recognizability of human facial expressions as rated by participants, but even outperform them, such as the “angry” and “sick” emojis (Fischer and Herbert, 2021; Cherbonnier and Michinov, 2021).

Despite these benefits, use of emojis in some settings can inhibit social functioning and relationships. For example, in professional settings, individuals who use emojis are perceived to be less competent and are therefore allocated fewer resources, which may hinder academic or career progress (Glikson et al., 2018). Additionally, when used inconsistently or inappropriately for a certain context, emojis can elicit cognitive dissonance for those who receive them (Adu-Mensah et al., 2024). Taken together, this prior work suggests that emojis have a context-dependent role in CMC, as they may enhance warmth and help clarify emotional valence during casual social interaction while simultaneously undermining professionalism and increasing discordance in more formal communication.

## Social anxiety and loneliness

Social anxiety is one of the most prevalent forms of psychopathology, with a rate of over 10% across the lifespan in the United States (Stein and Stein, 2008; Kessler et al., 2005). Primary symptoms include fear and avoidance of social situations, especially when an individual may be negatively evaluated (Chen et al., 2020). It often co-occurs with general anxiety, major depression, and substance use disorders and can predict future loneliness and low levels of social support (Leichsenring and Leweke, 2017; Ratnani et al., 2017).

Given that avoidance is a primary technique for reducing social anxiety, loneliness – characterized by a perceived lack of strong social relationships – often serves a reciprocal role with social anxiety to exacerbate social dysfunction (Eres et al., 2023; Motta, 2021; Mushtaq et al., 2014). Lonely individuals report feeling more apprehensive in social situations and are perceived as less competent during face-to-face communication – deficits in social skills which are closely linked to depression and anxiety (Moeller and Seehuus, 2019; Bell and Daly, 1985; Zakahi and Duran, 1982). Yet, while the relation between loneliness and social anxiety has been well-studied offline, its translation to CMC has seldom been examined and remains unclear.

## Social anxiety, loneliness, and digital communication

Some initial work has explored how symptoms of social anxiety relate to the receipt of CMC. For example, individuals with more severe social anxiety exhibit a larger negative interpretation bias in CMC, such that they perceive texts as being more negative than intended (Kingsbury and Coplan, 2016; Chen et al., 2020). The present study expands on the current literature through the use of an ecologically valid behavioral paradigm, which goes beyond prior studies on receipt and interpretation of emojis to target the sending of emojis and their use as a coping technique. Due to core features of social anxiety like fear of negative evaluation and intolerance of uncertainty (Winton et al., 1995; Whiting et al., 2014), sending texts may provoke distress similar to that of in-person communication in those with significant symptoms, especially because a lack of non-verbal cues in digital communication creates ambiguity of tone. Additionally, the Social Information Processing theory suggests that relationship and impression formation in CMC occur through increased textual cues to compensate for body language and facial expression (Walther, 2015). Thus, we posit that emoji usage may occur at a higher rate due to their ability to establish a clear interpretation and allow senders to regulate their distress by avoiding interpersonal conflict. Given that fear of negative evaluation is a hallmark symptom of social anxiety, use of emojis may reflect a compensatory strategy in CMC. Such a strategy may be potentiated among those with higher levels of loneliness, who have been shown to prefer CMC to in-person communication (Caplan, 2003) and may turn to an increased reliance on emojis as a substitute for face-to-face interaction. We aimed to clarify the relation between social anxiety, loneliness, and digital communication and expand on emoji use as a tool to offset distress from sending potentially ambiguous messages.

## Gender, social anxiety, and digital communication

There are significant gender differences in mental health symptoms and their relation to communication style. Social anxiety is more prominent in women with a lifetime prevalence rate of 15.5% for females and 11.1% for males, and some studies suggest that women feel more lonely than men (Chang et al., 2023; Nicolaisen and Thorsen, 2014). These differences apply to digital interactions as well, although the literature is comparatively sparse to that on in-person interactions. Some prior work has demonstrated that although males spend more time in front of screens, women spend more of their time engaged in communication with others while online (So et al., 2023; Twenge and Martin, 2020). In-person, women use more affiliative cues than men, including nodding, laughing, and exhibiting an open posture (Luxen, 2005). Furthermore, in digital communication, women tend to use a greater amount – and more diverse types – of emojis than men, depending on the social context in which CMC occurs (Chen et al., 2018; Koch et al., 2022).^1^ However, while many studies have explored the effects of gender CMC, few have investigated the way communication styles may differ in tandem with the interaction between gender and mental health symptoms. Given that women have an increased prevalence of social anxiety and loneliness, along with a stronger reliance on non-verbal cues in both in-person and digital communication, they may be more likely to use emojis as a compensatory strategy.

## Hypotheses

In this study, we investigated the role of social anxiety and loneliness in relation to emoji usage in the context of ambiguous text messages. Young adults with a range social anxiety symptoms and loneliness read vignettes that provided context for a text conversation. They were then asked to respond with a positively or negatively valenced text in which they could choose to include one of a number of different emojis, or to send the text without an emoji. We first sought to confirm that participants responded differently depending on valence in this novel measure of emoji use. The current literature on social anxiety, loneliness, and emoji use suggest a potential interplay of intolerance of uncertainty, compensatory strategies, and digital communication style (Whiting et al., 2014; Manganari, 2021; Kaye et al., 2016). We hypothesized (Hypothesis 1) that both higher levels of social anxiety and loneliness would be independently associated with greater emoji usage. We also hypothesized (Hypothesis 2) that the relation between social anxiety and emoji use would be stronger among participants with higher levels of loneliness. Furthermore, prior work demonstrates that women use emojis differently than men (Chen et al., 2018; Koch et al., 2022; Wirza et al., 2020; Kennison et al., 2025; Kennison et al., 2024). Thus, we hypothesized (Hypothesis 3) that this interaction would emerge in women, but not in men. Finally, fear of negative evaluation is a core feature of social anxiety (Winton et al., 1995). Therefore, we predicted (Hypothesis 4) that this relation would be potentiated in negative compared to positive contexts. Results clarify how digital communication patterns relate to mental health symptoms and lay a foundation for future work on the development of interventions for social anxiety and loneliness in emerging adults.

## Methods

### Participants

One hundred ninety-one participants were included in analyses (18–24 years of age, 64% female, 48% Asian, 37.2% white; see Table 1). All demographics were self-reported from standard multiple-choice categories. Three participants were excluded post-hoc from analyses due to being substantially older (>2.5 SDs) than the mean age of the sample (M ± SD = 20.10 ± 1.36; ages 25, 27, and 33). One participant was excluded post-hoc from analyses as they self-identified as transgender. While we recognize that this exclusion decreases the diversity and inclusivity of our sample, self-identification as being transgender is associated with differences in loneliness and communication compared to cisgender individuals (Heinz, 2018). Because of this, we chose to exclude the transgender participant due to the study’s focus on norms among cisgender males and females.

**Table 1:** Participant demographics.

Demographics	Total
*n*	191
Age	20.1 ± 1.36
Gender	
Female	63.9%
Male	36.1%
Race	
Asian	78 (40.8%)
White	71 (37.2%)
Hispanic/Latino	20 (10.5%)
Black	10 (5.2%)
Other/PNR	12 (6.3%)
Social anxiety	50.8 ± 26.7
Loneliness	40.8 ± 10.3
Depression	3.0 ± 2.2

PNR, prefer not to respond.

Data collection took place asynchronously and online from 04/03/2018 to 05/04/2018. Participants were recruited through a student research participation pool at a public university in the Northeast United States. Participants were required to be fluent in English, have reliable access to a computer and internet connection, and be currently enrolled as students. No minors were included in this study. Although there were no financial incentives, students who completed all parts of the study received course-related research credit. All participants provided written consent (obtained digitally) to study procedures, which were approved by the University’s Internal Review Board. Participants took 21.1 ± 14.1 min on average to complete the survey, which they could pause and resume at any time. There was no maximum time threshold, and attention checks were not implemented.

### Study procedures

Qualtrics was used for all self-paced data collection. Participants responded to 23 CMC vignette prompts and completed a battery of questionnaires on constructs of social anxiety, loneliness, and depression. Reverse-coded items were scored properly, and each construct was quantified as the sum of responses for the purposes of analysis. Rather than applying a cutoff for clinical significance, all scores were treated as a continuous variable to facilitate use of linear regression models and more nuanced analyses.

#### Social anxiety

Social anxiety symptoms were measured by the self-reported Liebowitz Social Anxiety Scale (LSAS), a 24-item survey measuring fear and avoidance of different social situations (Liebowitz, 1987). Items were measured on a 4-point scale ranging from “no fear/avoidance” to “severe fear/avoidance,” and participants were asked to base their ratings on the degree to which hypothetical scenarios may have affected them had they occurred during the past week (Liebowitz, 1987). The LSAS has high internal consistency within this sample (Cronbach’s alpha = 0.96).

#### Loneliness

Loneliness was measured by the self-reported UCLA Loneliness Scale, a 20-item survey measuring general feelings of loneliness (Russell et al., 1978). Items were measured on a 4-point scale ranging from “I never feel this way” to “I often feel this way,” and participants were asked to base their ratings on their overall life experiences (Russell et al., 1978). The UCLA Loneliness Scale had high internal consistency within this sample (Cronbach’s alpha = 0.92).

#### Depression

Although depression is not a primary construct of interest, symptoms often co-occur with both social anxiety and loneliness, thus it was important to assess and control for it in analyses (Ratnani et al., 2017). Depression was measured by the self-reported DSM-5 Cross Cutting Symptoms Depression Subscale, a 2-item subscale of the full 23-item questionnaire that focus specifically on depressive symptoms (Narrow et al., 2013). Although two items alone may not fully capture depression severity, due to time constraints for the full survey, we chose to use this scale rather than a longer, more clinically validated scale to improve participant experience. Items were measured on a 5-point scale ranging from “not at all” to “nearly every day,” and participants were asked to base their ratings on their experiences in the last 2 weeks (Narrow et al., 2013). The DSM-5 Cross Cutting Symptoms Depression Subscale has moderately high internal consistency within this sample (Cronbach’s alpha = 0.76).

#### CMC vignette task

**Figure 1:**
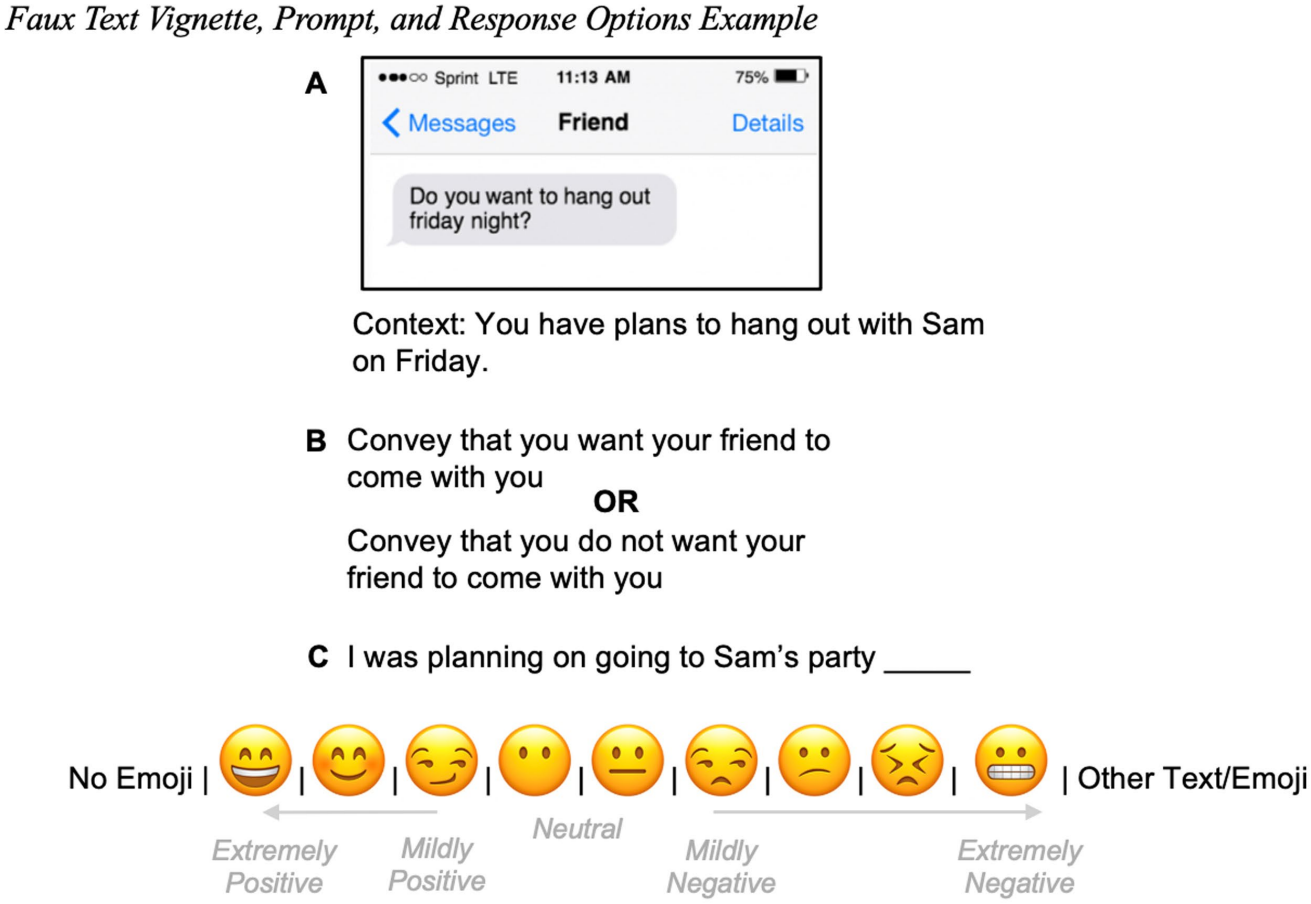
**(A)** Participants viewed one of 12 possible vignettes, **(B)** followed by an instruction to convey either positively or negatively valanced meaning. **(C)** Participants then had the option to respond without an emoji, with one to nine preselected emojis, or an additional emoji of their choice. Participants did not see the text in gray portraying the valence coding of each emoji for analyses purposes.

Stimuli, prompts, and valence distinctions were adapted from prior work measuring emoji usage (Kingsbury and Coplan, 2016). In this task, participants were presented with 23 vignettes of what appeared to be a smartphone message from a friend, along with additional context if necessary (Figure 1A). After reading each message, participants were instructed to convey either a positive or negative sentiment (Figure 1B). They were given the option of sending an ambiguous pre-written response as is, including one of nine emojis ranging from happy to displeased, or adding additional text or emoji(s) (Figure 1C). These nine emojis were selected to provide a unidimensional representation of happiness and displeasure without, additional features, such as tears or sweat drops, that may imply emotions beyond this dimension. This task was intended to mimic real-world texting behavior in different social contexts while also maintaining scientific robustness.

All participants saw the same 23 vignettes, but valence order was counterbalanced; participants were randomly assigned either to respond to the first 12 vignettes negatively and to the other 11 positively (*N* = 98) or vice versa (*N* = 93; see Supplementary Table S1 for participant demographics by survey group and Supplementary Table S3 for a list of all vignettes/prompts). Vignettes order within valence were not randomized. This yielded 46 unique vignette/prompts combinations.

### Data analysis

All analyses were performed in R (version 4.4.1); specific packages are noted when appropriate.

#### Factor analysis for CMC vignette task

The CMC Vignette Task is novel. Therefore, it was important to perform a manipulation check to confirm that participants used different types of emojis to respond to prompts that researchers designed to be positive as compared to negative in valence. Moreover, to reduce the number of statistical tests being performed and increase reliability of results, we sought to analyze emoji usage based on valence (i.e., positive or negative), rather than on a prompt-by-prompt basis. A factor analysis enabled us to confirm that a subset of prompts loaded onto a “positive” or a “negative” factor, in that they did, in fact, elicit generally positive or negative emoji responses. Prompts that did not load onto these primary factors were omitted from primary hypothesis testing.

As shown in Figure 1C, emoji responses were defined as being mildly to extremely positive or negative, and the “No Emoji” and “Other Text/Emoji” options were classified as neither positive nor negative. Emoji responses were converted to a numeric scale for factor analysis, with 1 representing extremely positive emojis and 10 representing extremely negative emojis. Participant responses for the “other text/emoji” option containing an emoji other than the nine provided in the scale were not coded for valence and were not included in factor analysis.

We also performed an additional manual exploratory review of the most common responses to each vignette; this allowed for the identification of potential secondary valence categories and facilitated the exclusion of vignettes that did not align with any factor. Factor analyses were performed with *R*’s “psych” package (Revelle, 2025).

#### Relation among emoji usage, social anxiety, and loneliness by gender and social context

Given that social anxiety, loneliness, and depression often co-occur, we performed confirmatory analyses for colinearly among variables. To do this, Pearson product–moment correlation analyses were performed between DSM-5 Cross Cutting Symptoms Depression Subscale scores, UCLA Loneliness Scale scores, and Liebowitz Social Anxiety Scale scores for both males and females. Depression was correlated with loneliness (*r*_female_ = 0.473, *p* < 0.001; *r*_male_ = 0.528, *p* < 0.001) and social anxiety (*r*_female_ = 0.412, *p* < 0.001; *r*_male_ = 0.371, *p* = 0.005).

Based on these analyses, depression was then residualized out from both social anxiety and loneliness to account for collinearity using *R*’s “umx” package (Bates et al., 2019). Total emoji use was selected as the dependent variable and was expressed as a percentage. This value was calculated by adding together the number of responses with an emoji regardless of valence and dividing by the total number of prompts. If a participant chose to enter an emoji that was not one of the nine in the scale provided using the “other text/emoji” option, we also include this as a response with an emoji. To test Hypotheses 1, 2, and 3, two separate regressions were performed – one for males, one for females – using social anxiety, loneliness, and their interaction as independent variables to test whether they predicted total emoji usage. This was done because the relatively small sample size did not provide sufficient power to test for a three-way interaction between social anxiety, loneliness, and gender.

For significant results, additional analyses tested Hypothesis 4, whether effects varied by valence categories revealed by factor analysis and our exploratory review of the vignettes/prompts. To facilitate interpretation, follow-up simple slopes analyses using *R*’s “reghelper” package were performed on participants grouped by levels (M ± 1SD) of social anxiety and plotted using *R*’s “ggplot2,” “interactions,” and “jtools” packages (Hughes and Beiner, 2023; Wickham et al., 2025; Long, 2024a; Long, 2024b). Normality of residuals was evaluated visually using Q–Q plots rather than formal significance tests, which can be overly sensitive in moderate samples. Linearity and homoscedasticity were assessed via residual-vs-fitted plots. Given the known correlation between social anxiety and loneliness, multicollinearity was assessed with predictor-level variance inflation factors (VIF) and tolerance, using conservative criteria (VIF < 5; tolerance > 0.20).

## Results

### Factor analysis

The exploratory factor analysis of the 46 vignettes was conducted on data from 191 participants using the minimum residual method of extraction and the default varimax method of oblique rotation. Items with factor loadings >0.30 were considered nonsignificant (see Supplementary Table S2 for scree plot). This analysis suggested that prompts loaded onto two factors: positive social context and negative social context. Overall, vignettes prompting participants to convey a positively valanced response yielded more positive emoji use and loaded uniquely on the positive factor (*N* = 15) and vignettes prompting participants to convey a negatively valanced response yielded more negative emoji use and loaded uniquely on the negative factor (*N* = 16). A subset of prompts did not load onto either factor (*N* = 8) and were omitted from analyses.

An exploratory review of prompts with significant loadings was conducted in order to further identify unique ways in which participants responded to the vignettes and prompts. Unexpectedly, this revealed a subset of prompts that loaded onto both factors (*N* = 7) such that both positive and negative emojis were used regardless of the vignettes’ intended valence. These prompts were separated into an exploratory grouping of “ambiguous” vignettes. The exploratory review also revealed that among negative prompts, a subset (*N* = 5) yielded more extremely negative emoji responses. These prompts were separated into another exploratory grouping of “very negative” vignettes. There were no differences in responses used for positive vignettes. This resulted in a total of two factors (positive and negative) and two exploratory groupings (very negative and ambiguous) which yielded four additional exploratory regression models (very negative and ambiguous, by gender). Model assumptions were validated using the same methodology as the primary valence categories. See Table 2 for more information on these exploratory groupings, as well as a full list of vignettes and their loadings, can be found in Supplementary Tables S3–S5).

**Table 2:** Final valence categories.

Valence	Total # vignettes
Positive valence	15
Negative valence	16
Very negative	5
Ambiguous valence	7
Total	38

### Relation among emoji usage, social anxiety, and loneliness by gender and social context

See Table 3 for statistics for all main effects and interactions. Visual inspection of residual and Q-Q plots indicated that linearity and normality assumptions were reasonably met for all models, with only minor deviations in the tails. Some modest heteroscedasticity was observed, particularly in Total and Negative Emoji Use models. See Supplementary Table S6 for plots and full VIF statistics. No major violations of independence were evident. VIF values were all 1, reflecting the residualization of predictors to address collinearity.

**Table 3:** Regression results.

Valence & variable	Females		
	*R*^2^, CI 95%	*β*, CI 95%	*p*
Total	0.072 [0.01, 0.20]		
Loneliness		−0.101 [−0.18, −0.02]	0.015*
Social anxiety		0.023 [−0.00, 0.05]	0.094
Interaction		0.004 [0.00, 0.01]	0.013*
Positive	0.112 [0.02, 0.25]		
Loneliness		−0.067 [−0.00, −0.03]	0.000***
Social anxiety		0.012 [−0.00, 0.02]	0.061
Interaction		0.001 [0.00, 0.00]	0.037*
Negative	0.001 [0.00, 0.09]		
Loneliness		−0.023 [−0.07, 0.03]	0.376
Social anxiety		0.004 [−0.01, 0.02]	0.620
Interaction		0.002 [−0.00, 0.00]	0.103
Very negative	0.063 [0.00, 0.19]		
Loneliness		−0.023 [−0.00, 0.01]	0.017*
Social anxiety		−0.000 [−0.00, −0.00]	0.934
Interaction		0.001 [0.00, 0.00]	0.031*
Ambiguous	0.003 [0.00, 0.10]		
Loneliness		−0.012 [−0.04, 0.02]	0.483
Social anxiety		0.007 [−0.00, 0.02]	0.179
Interaction		0.000 [−0.00, 0.00]	0.147

Valence & variable	Males		
	*R*^2^, CI 95%	*β*, CI 95%	*p*
Total	0.044 [0.00, 0.24]		
Loneliness		−0.025 [−0.19, 0.14]	0.769
Social anxiety		−0.069 [−0.13, −0.00]	0.039*
Interaction		0.003 [−0.00, 0.01]	0.440
Positive	−0.024 [0.00, 0.12]		
Loneliness		−0.032 [−0.11, 0.04]	0.403
Social anxiety		−0.006 [−0.03, 0.02]	0.674
Interaction		0.001 [−0.00, 0.00]	0.414
Negative	0.059 [0.00, 0.26]		
Loneliness		−0.008 [−0.08, −0.07]	0.839
Social anxiety		−0.033 [−0.06, −0.00]	0.027*
Interaction		0.002 [−0.00, 0.01]	0.312
Very negative	0.128 [0.01, 0.34]		
Loneliness		0.016 [−0.01, 0.05]	0.278
Social anxiety		−0.018 [−0.03, −0.01]	0.003**
Interaction		0.000 [−0.00, 0.00]	0.268
Ambiguous	0.120 [0.00, 0.34]		
Loneliness		0.014 [−0.03, 0.06]	0.562
Social anxiety		−0.030 [−0.05, −0.01]	0.002**
Interaction		0.000 [−0.00, 0.00]	0.893

**Figure 2:**
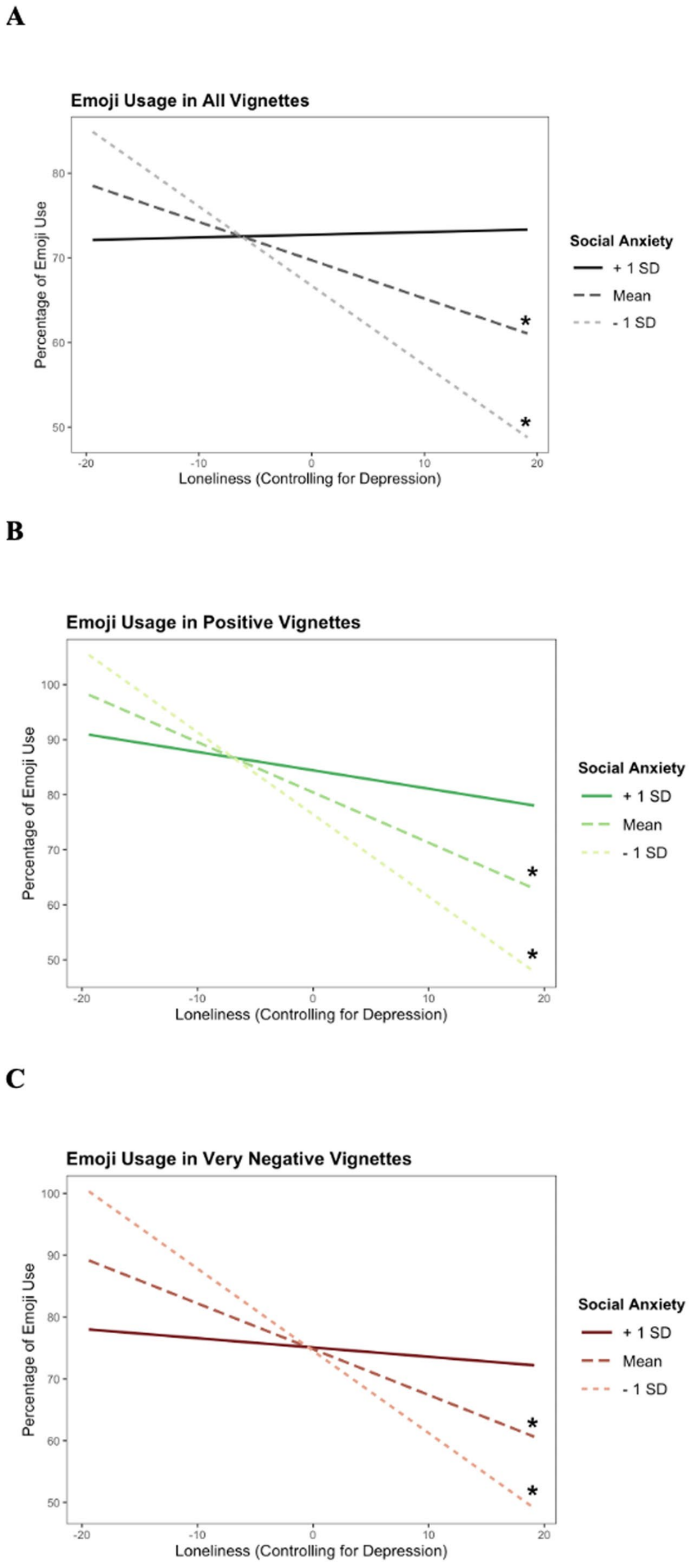
**(A)** Simple slopes of emoji use in all vignettes—female only. **(B)** Simple slopes of emoji use in positive vignettes—female only. **(C)** Simple slopes of emoji use in very negative vignettes—female only.

Hypothesis 1, that higher levels of social anxiety and loneliness would be independently associated with greater emoji usage, was not supported across all participants. Hypothesis 2, that among participants with higher levels of loneliness, more severe symptoms of social anxiety would be associated with greater emoji use, was also not supported. However, among females, main effects of loneliness emerged for total emoji use and emoji use in positive and very negative contexts such that higher levels of loneliness were associated with lower levels of emoji use. These main effects were qualified by loneliness-by-social anxiety interactions (Hypothesis 3). Simple slopes analyses demonstrated that loneliness attenuated total emoji use (Figure 2A) and emoji use in positive (Figure 2B) and very negative (Figure 2C) contexts (Hypothesis 4) among those with average (total: *β* = −0.452, *p* = 0.038; positive: *β* = −0.914, *p* < 0.001; very negative: *β* = −0.702, *p* = 0.035) and low (total: *β* = −0.936, *p* = 0.003; positive: *β* = −1.494, *p* = 0.00; very negative: *β* = −1.330, *p* = 0.009) social anxiety. These effects were not observed among those with high social anxiety (total: *β* = 0.031, *p* = 0.915; positive: *β* = −0.334, *p* = 0.281; very negative: *β* = −0.150, *p* = 0.750). There were no main effects of social anxiety on emoji use. See Supplementary Table S7 for full simple slopes statistics.

Among males, there were no main effects of loneliness or loneliness-by-social anxiety interactions (Hypothesis 3). Main effects of social anxiety did emerge for total emoji use as well as emoji use in negative, very negative, and ambiguous contexts (Hypothesis 4) such that lower levels of social anxiety were associated with higher levels of emoji use.

## Discussion

Little research has been conducted on the relation between social anxiety, loneliness, and emoji usage in males and females. The present study addressed this using experimentally manipulated text messages and prompts to measure participants’ emoji usage in different social contexts. We identified four distinct patterns of emoji use, corresponding to four contexts with varying social valences (positive, negative, very negative, and ambiguous). Our results demonstrate that the interaction of social anxiety and loneliness were significantly related to emoji usage, but that this relation varied depending on context and gender. To our knowledge, this study is the first to demonstrate this nuanced relation. Despite the modest effect size and sample diversity, these results still have important preliminary implications considering the widespread nature of digital communication and the large percentage of young adults that use CMC regularly (Gottfried, 2024).

Individual differences in social anxiety and loneliness relate to distinct patterns of emoji use in males and females, but not in the hypothesized direction. For example, while social anxiety predicts lower emoji usage in males overall and in negative and ambiguous social context, this effect is not demonstrated in females. Conversely, while loneliness and the loneliness-by-social anxiety interact to predict lower emoji usage in females overall and in positive or very negative social contexts, this effect is not demonstrated in males. Among females, lower emoji use emerged when higher levels of loneliness occurred in conjunction with moderate or less severe symptoms of social anxiety. This is inconsistent with our hypothesis that more severe social anxiety and loneliness would yield greater emoji use – an effect we expected to be potentiated in those with high levels of both social anxiety and loneliness. Results more broadly support prior literature suggesting differences in emoji usage across social context (Chen et al., 2018; Koch et al., 2022; Wirza et al., 2020; Kennison et al., 2025; Kennison et al., 2024).

Additionally, gender-related differences in communication style have real-world implications that may impact how competently individuals are perceived and thus influence the resources they are given (Glikson et al., 2018). For example, lack of significant results in males may be due to underpowered subgroup analysis; however, this could also be interpreted as a reflection of social norms surrounding masculinity and the idea that men should not openly express emotion. Similarly, the significant results for women may reflect social norms surrounding femininity and the idea that women are to preserve softness and non-conflict. These gender differences may be particularly important in a post-COVID-19 world, where reliance on technology for social interaction is heightened both for leisure and business (DeFilippis et al., 2022; Saud et al., 2020; Afridi et al., 2023). As the use of digital media as a means of seeking social connection and support increases, so do the potential adverse effects on mental health (Draženović et al., 2023). This study sheds light on possible avenues for expression of mental health symptoms through digital communication and may have important applications in identifying those most in need of clinical care.

Despite its strengths, the current study does present limitations. First and foremost, the cross-sectional nature of this study fails to establish causal inference. The prompts used to measure emoji usage were only hypothetical and did not measure participants’ real-world emoji usage in texts and direct messaging platforms. Additionally, these prompts were only meant to elicit positive or negative valence and did not account for the wider range of emotional valence which can occur during communication. Factor analysis and our exploratory valence categories were meant to expand our understanding of the range of contexts participants may have tried to convey, but these limitations may impact results nonetheless. The emoji choices for this study also focused only on those portraying happy or displeased emotions, whereas the most commonly used emojis tend to be those of humor, love, or sadness (Chen et al., 2018; Wirza et al., 2020). The novelty of this study may explain why it fails to replicate some previous findings. However, other limitations associated with the sample may have also contributed to discrepancies, including small size, limited ethnic/racial diversity, predominantly female (64%), and, on average, moderate levels of social anxiety. Future studies should investigate emoji use in a more natural setting (e.g., real text data, social media posts, etc.), include a wider range of emojis that allow participants to express different emotions, or employ methods such as ecological momentary assessments and longitudinal data collection. Further research may also benefit from studying participants with greater gender and racial/ethnic diversity and assessing culture influences how emojis are interpreted and used.

The methods used in this study, following replication with larger and more diverse samples, may help provide preliminary insights for future experimental research on digital communication style, mental health, and gender. The nuanced relations between social anxiety, loneliness, gender, emoji use may have potential implications in the development of novel interventions aimed at identify individuals that may gain most benefit from psychosocial interventions, especially due to the high levels of reliance on CMC among emerging adults (Gottfried, 2024). For example, a shift from frequent use of positive emojis to negative or to more neutral, affectively flat emojis may indicate a decline in well-being or an increase in depressive symptoms. Overall, given the integral role of CMC in our evolving social landscape, assessing how new forms of communication influence social behavior is imperative to furthering our understanding of mental health in the coming years.

## Data Availability

The datasets presented in this study can be found in online repositories. The names of the repository/repositories and accession number(s) can be found below: https://osf.io/4w6gd/.

## Footnotes

^1^While the “tears of joy” emoji is the most commonly used among both genders, the second-most common emoji is “loudly crying” for women and “folded hands” for men (Wirza et al., 2020). Other studies have also demonstrated that the relation between personality traits and emoji use differ for men and women; while emoji use is associated with low openness to experience for both genders, narcissism is associated with the highest emoji use in women, while Machiavellianism and neuroticism is associated with the highest use in men (Kennison et al., 2025; Kennison et al., 2024).
