# Supplementary material for: Social anxiety and emoji use: gender differences and the role of loneliness in digital communication among college students
Source: Front Psychol. 2025 Oct 23;16:1626509. doi: 10.3389/fpsyg.2025.1626509 (PMC12588911; doi:10.3389/fpsyg.2025.1626509)
Supplement: Supplementary file 1 [file Table_1.docx]

**S1 Table: Participant Demographics**

|  | **Survey Group 1** | | **Survey Group 2** |
| --- | --- | --- | --- |
| n | 98 | 93 | |
| Age  M ± SD  Gender  Female  Male | 20.1 ± 1.34  65 (66.3%)  33 (33.7%) | 20.2 ± 1.38  57 (61.3%)  36 (38.7%) | |
| Race  Asian  White  Hispanic/Latino  Black  Other/PNR | 39 (39.8%)  37 (37.8%)  13 (13.3%)  3 (3.1%)  6 (6.1%) | 39 (41.9%)  34 (36.6%)  7 (7.5%)  7 (7.5%)  6 (6.5%) | |
| Social Anxiety |  |  | |
| M ± SD | 48.1 ± 29.5 | 53.8 ± 22.9 | |
| Loneliness |  |  | |
| M ± SD | 40.9 ± 11.1 | 40.7 ± 9.5 | |
| Depression |  |  | |
| M ± SD | 3.0 ± 2.3 | 3.0 ± 2.2 | |

Note: PNR = Prefer Not to Respond
