# Supplementary figures and images for: Social anxiety and emoji use: gender differences and the role of loneliness in digital communication among college students
Source: Front Psychol. 2025 Oct 23;16:1626509. doi: 10.3389/fpsyg.2025.1626509 (PMC12588911; doi:10.3389/fpsyg.2025.1626509)

**S2 Fig: Factor Analysis Scree Plot**


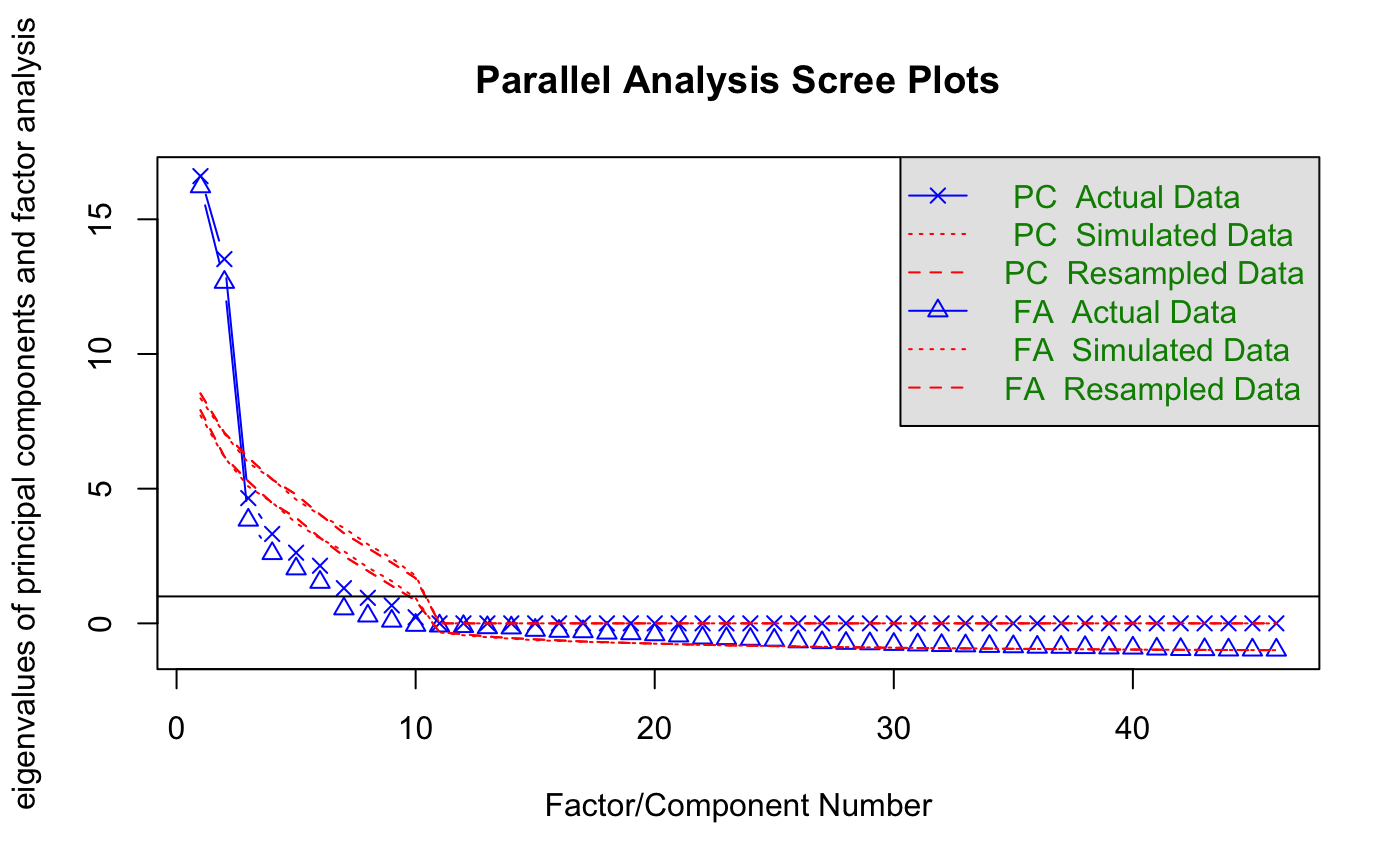

Supplement: Supplementary file 2 [file Table_2.docx]

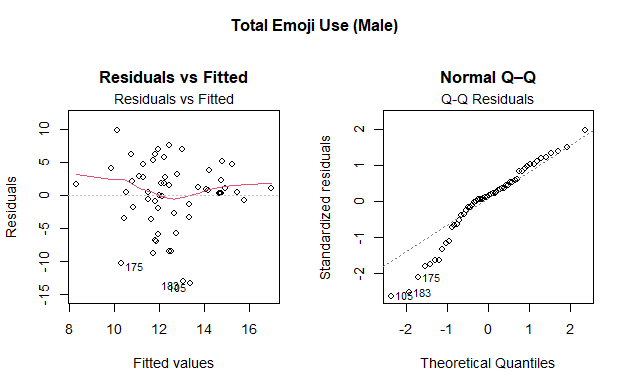

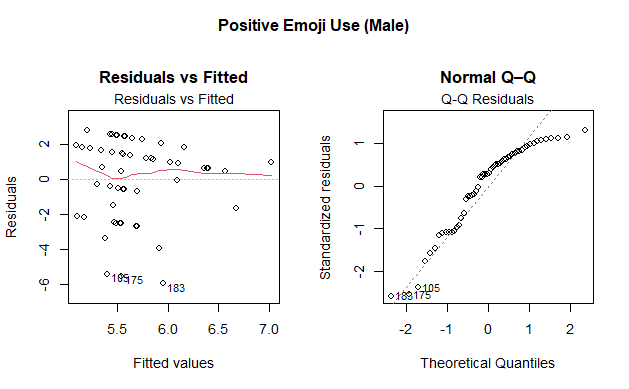

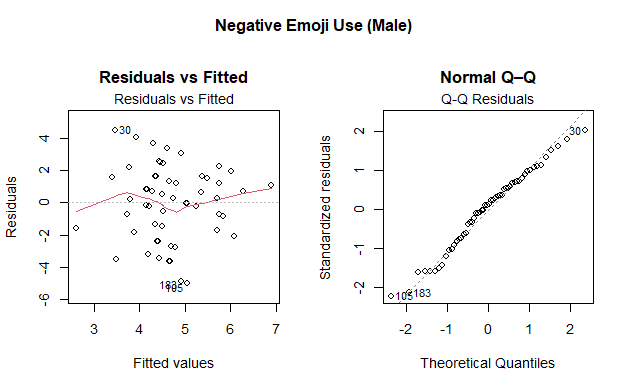

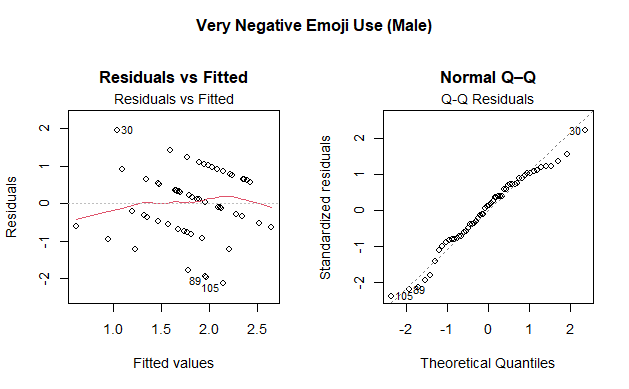

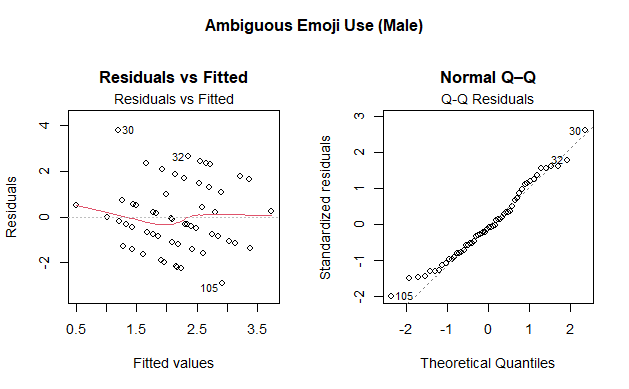

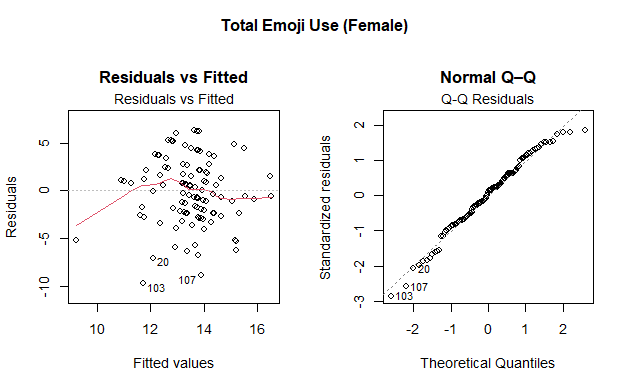

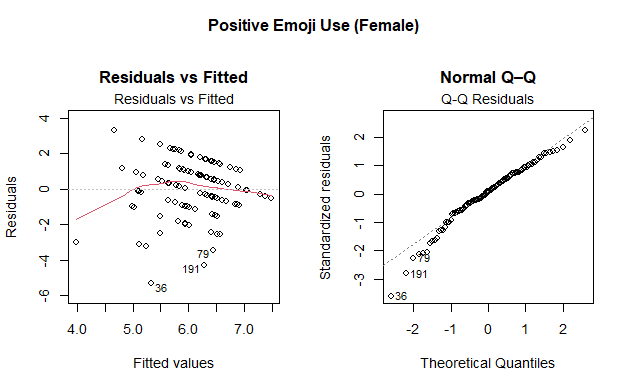

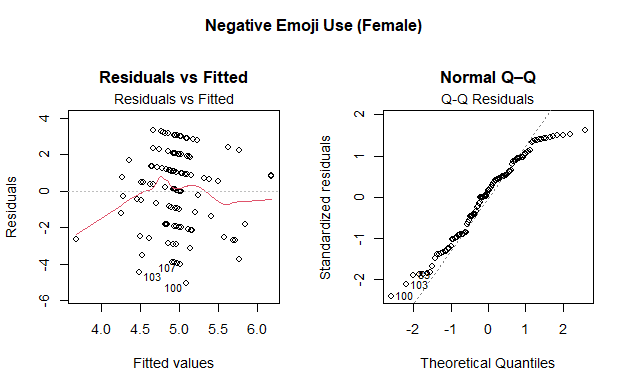

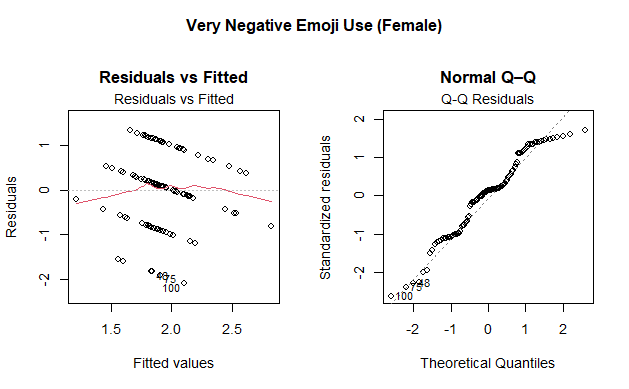

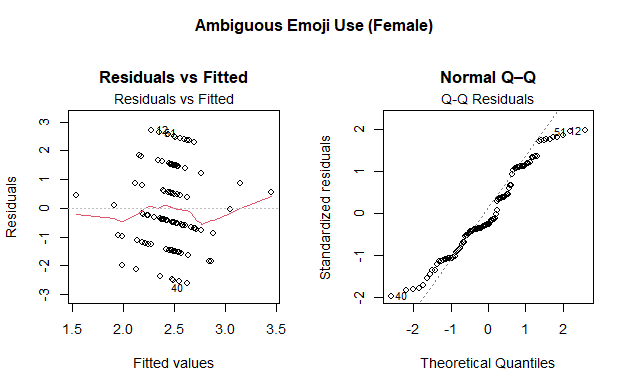

Supplement: Supplementary file 6 [file Table_6.docx]
