# Supplementary material for: Social anxiety and emoji use: gender differences and the role of loneliness in digital communication among college students
Source: Front Psychol. 2025 Oct 23;16:1626509. doi: 10.3389/fpsyg.2025.1626509 (PMC12588911; doi:10.3389/fpsyg.2025.1626509)
Supplement: Supplementary file 3 [file Table_3.docx]

**S3 Table: Factor Analysis Results**

| **Item** | **Factor 1 (Positive)** | **Factor 2 (Negative)** |
| --- | --- | --- |
| “Want to hang out on Thursday?” Convey to your friend that you don't really want to hangout. | 0.06101693 | 0.959576165* |
| “Hey, what are you up to tonight?” Convey that you do not want to hang out with your friend tonight. | 0.28750178 | 0.932551992* |
| “Are you free to talk?” Convey to your friend that you do not want to talk to them. | -0.16849204 | 0.929086298* |
| “So what do you want to do today?” Convey that you do not want to hangout with your friend today. | 0.18002239 | 0.915022839* |
| “Did you get my message?” Yesterday, your friend sent you a long message, and you haven't responded. They texted you to follow up. Convey to your friend that you just didn't want to respond to them. | -0.18259063 | 0.910993222* |
| “What happened before was so funny!” Convey to your friend that you didn't think it was funny, and are brushing them off. | 0.06882602 | 0.903765055* |
| “Are you there?” A friend calls you and you don't answer. Convey to your friend that you don't want to talk right now. | -0.24533945 | 0.893847733* |
| “Sorry I missed your call, what’s up?” Convey that you have something bad to tell your friend. | -0.17496111 | 0.875583958* |
| “We are in the same class together!” Convey to your friend that you don't really want to see them in class. | 0.287216 | 0.861053116* |
| “Want to get some dinner before the concert?” Convey to your friend that you don't really want to have dinner with them. | -0.10833757 | 0.806840247* |
| *“Did you get my message?” Yesterday, your friend sent you a long message, and you haven't responded. They texted you to follow up. Convey to your friend that you did not get the message.* | *-0.3827753** | *0.771721388** |
| “Hey, what are you up to?” You are already hanging out with your friend Jamie. Convey to your friend that you do not want them to join you and Jamie. | 0.08749761 | 0.761677063* |
| “OMG I just got offered my dream job!” Convey to your friend that you don't really care. | 0.39078233* | 0.744126384* |
| *“Are you there?” A friend calls you and you don't answer. Convey to your friend that you are busy right now.* | *-0.4305185** | *0.713523677** |
| “I was so nervous about that presentation today. How did I do?” Convey that you did not like your friend's presentation. | 0.74855614* | 0.577717224* |
| “Are you going to take my advice?” You have a problem and texted your friend for advice. After they say what they think you should do, they message you the above. Convey to your friend that you think they gave bad advice. | -0.41414901* | 0.56726497* |
| “I started this fun new hobby.” A friend messaged you to tell you about a new hobby they started. Convey to your friend that you are being sarcastic. | 0.40383109* | 0.521244483* |
| “Sorry but I’m too tired to hang out now.” Convey to your friend that you are mad at them for canceling. | -0.42495877* | 0.518424011* |
| “Are you free to talk?” Convey to your friend that you are busy but want to talk later. | 0.33334946* | 0.511590199* |
| “Sorry but I’m too tired to hang out now.” Convey to your friend that you understand that they had to cancel. | 0.51226566* | 0.472107774* |
| “Do you want to hang out Friday night?” You had plans to go to your friend Sam's party Friday night. Convey that you do not want your friend to come to Sam's with you. | -0.35390958* | 0.461636529* |
| “Hey can I borrow your notes from the class I missed?” Convey to your friend that you do not want to let them copy your notes. | -0.36708267* | 0.394269361* |
| *“Can you save me a seat in class?” Convey that you want to sit with the friend who messaged you, not Alex.* | *-0.62144941** | *0.392074333** |
| “Can you save me a seat in class?” Convey that you want to sit with Alex, not the friend who messaged you. | -0.39934931* | 0.352236856* |
| “Want to hang out on Thursday?” Convey to your friend that you have to check your schedule. | 0.79346039* | 0.330806797* |
| “Are you going to take my advice?” You have a problem and texted your friend for advice. After they say what they think you should do, they message you the above. Convey to your friend that you think they gave good advice. | 0.85579345* | 0.318582495* |
| *“Is it ok if I bring someone with me to your party?” Convey that you do not want your friend to bring someone to your party.* | *-0.54125988** | *0.292893014* |
| “Hey can I borrow your notes from the class I missed?” Convey to your friend that you do not mind lending them your notes. | 0.86275016* | 0.27934529 |
| *“I heard about last night.” The day after attending a party, your friend messages you. Convey to your friend that something embarrassing happened to you at the party.* | *-0.40493862** | *0.25641759* |
| *“Did you end up finishing our project?” You have been working on a group project with your friend. Convey to your friend that you are mad at them for not doing more work on your project.* | *-0.3581393** | *0.220405245* |
| “Hey, what are you up to tonight?” Convey that it would be ok for your friend to come over to hangout with you. | 0.83365563* | 0.17744427 |
| “What happened before was so funny!” Convey to your friend that you thought it was funny. | 0.67160731* | 0.175930197 |
| “Hey, what are you up to?” You are already hanging out with your friend Jamie. Convey to your friend that you want them to join you and Jamie. | 0.96553606* | 0.086808077 |
| “So what do you want to do today?” Convey that you do not mind what you do with your friend today. | 0.97047669* | 0.065886545 |
| “Is it ok if I bring someone with me to your party?” Convey that it is fine if your friend brings someone. | 0.92446146* | 0.063793917 |
| “Sorry I missed your call, what’s up?” Convey that you have something exciting to tell your friend. | 0.94427535* | 0.017157797 |
| *“I saw an interesting picture of you.” Convey to your friend that you won't like the photo.* | *-0.7335717** | *0.005760315* |
| “Want to get some dinner before the concert?” Convey to your friend that you want to have dinner with them. | 0.97319323* | -0.041622548 |
| I saw an interesting picture of you.  Convey to your friend that you will like the photo. | 0.46552051* | -0.074453359 |
| *“I heard about last night.” The day after attending a party, your friend messages you. Convey to your friend that something interesting happened at the party.* | *0.04736309* | *-0.14131987* |
| “Do you want to hang out Friday night?” You had plans to go to your friend Sam's party Friday night. Convey that you want your friend to come with you. | 0.5345126* | -0.141726889 |
| “I was so nervous about that presentation today. How did I do?” Convey that you liked your friend's presentation. | 0.91947533* | -0.185774619 |
| “I started this fun new hobby.” A friend messaged you to tell you about a new hobby they started. Convey to your friend that you think it is interesting. | 0.94853631* | -0.192503074 |
| “Did you end up finishing our project?” You have been working on a group project with your friend. Convey to your friend that you are excited to have finished the project. | 0.76731695* | -0.232115163 |
| “OMG I just got offered my dream job!” Convey to your friend that you are happy for them. | 0.81024536* | -0.235767523 |
| “We are in the same class together!” Convey to your friend that you will be happy to see them. | 0.80207765* | -0.250903862 |

*Note: * = p<0.05. Color coding represents factor/subfactor: red = negative, dark red = very negative, yellow = cross valence, dark grey = no significant loadings, blue = no significant negative loading, green = positive (see Supplemental Figures 2-7). Italic items with loadings shaded light grey were ultimately removed for analyses.*
