# Supplementary material for: Social anxiety and emoji use: gender differences and the role of loneliness in digital communication among college students
Source: Front Psychol. 2025 Oct 23;16:1626509. doi: 10.3389/fpsyg.2025.1626509 (PMC12588911; doi:10.3389/fpsyg.2025.1626509)
Supplement: Supplementary file 4 [file Table_4.docx]

**S4 Table: Final Valence Categories**

|  | **Survey Group 1** | **Survey Group 2** |
| --- | --- | --- |
| Positive Valence | 7 | 8 |
| Negative Valence | 8 | 8 |
| Very Negative | 3 | 2 |
| Ambiguous Valence | 5 | 2 |
| **TOTAL** | 20 | 18 |
