# Supplementary material for: Social anxiety and emoji use: gender differences and the role of loneliness in digital communication among college students
Source: Front Psychol. 2025 Oct 23;16:1626509. doi: 10.3389/fpsyg.2025.1626509 (PMC12588911; doi:10.3389/fpsyg.2025.1626509)
Supplement: Supplementary file 5 [file Table_5.docx]

**S5 Appendix: Factor Explanations**

*Positive Factor: Vignettes Intended to Convey Positive Meaning with High Frequency of Happy Emojis and Low Frequency of Negative Emojis*

*
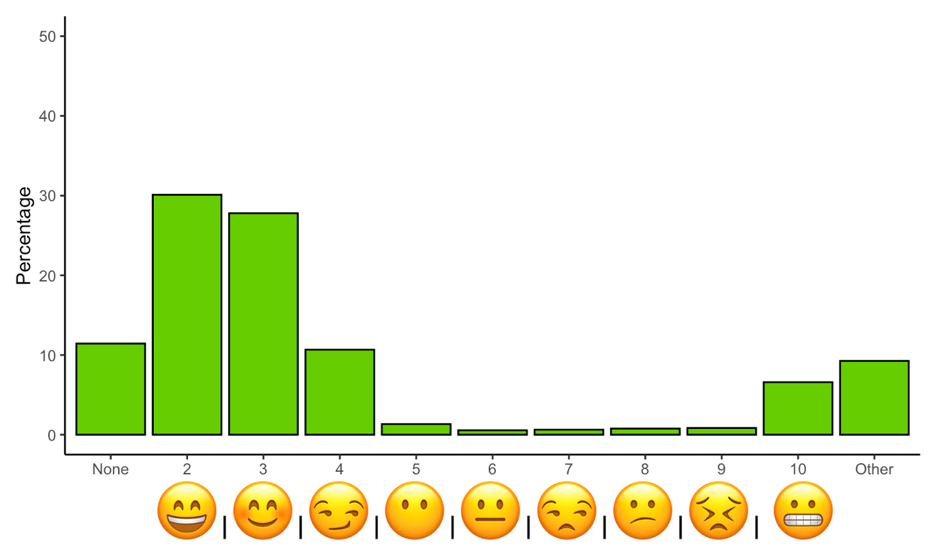
*

For 15 items where participants were instructed to convey positive meaning, emojis 2 through 3 – the happiest emoji options were most frequently used . This is what we expected for vignettes prompting participants to convey positive meaning. Therefore, these 15 items were categorized as the “positive factor”.

*Negative Factor: Vignettes Intended to Convey Negative Meaning with Moderate Frequency of Displeased Emojis and Low Frequency of Happy Emojis*

***
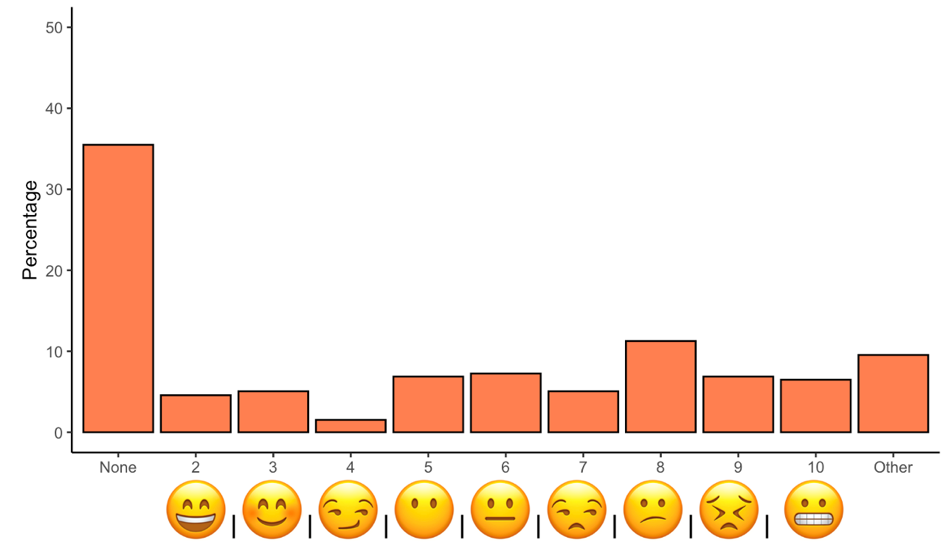
***

For 11 items where participants were instructed to convey negative meaning, the most frequent responses were either the “no emoji” option or emojis 7 through 9 – the most displeased emoji options. This is what we expected for vignettes prompting participants to convey negative meaning. Therefore, these 11 items were categorized as the “negative factor”.

*Very Negative Factor: Vignettes Intended to Convey Negative Meaning with High Frequency of Displeased Emojis and Very Low Frequency of Happy Emojis*


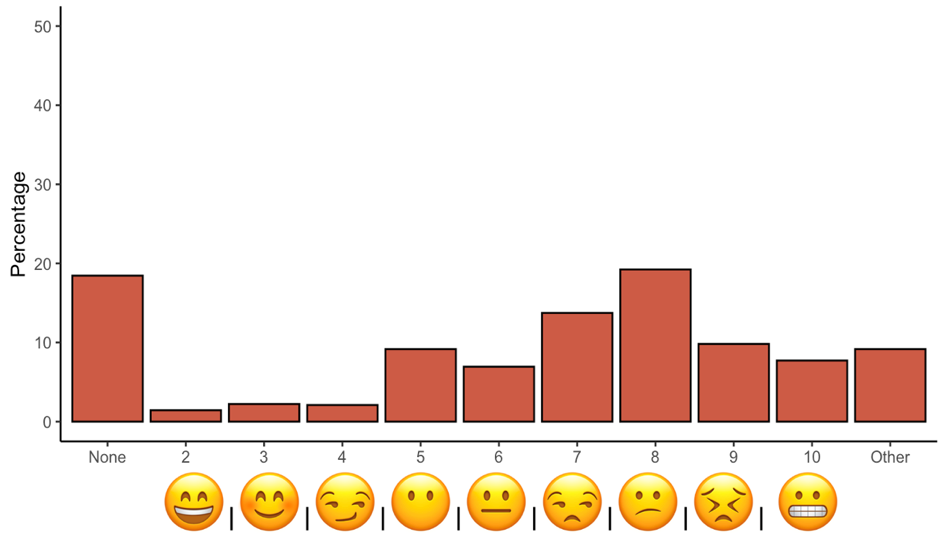


For 5 items intended to elicit negative responses and 3 items intended to elicit positive responses, responses of “no emoji” were less frequent and emojis 7 through 10 – the most displeased emojis – were more frequent than the vignettes that loaded only positively onto the negative factor. Because they did not load similarly to the other positive vignettes, we excluded the 3 items intended to elicit positive responses from future analyses. Additionally, the 5 items in this pattern that were intended to elicit negative responses elicited negative emojis at a higher rate and positive responses were used at a lower rate compared to other negative vignettes, leading to a distinct distribution. Therefore, these items were included in future analyses but separated into an exploratory “subfactor” called “very negative” to delineate the differences in loadings.

*Cross Valence Factors: Vignettes Intended to Convey Either Negative or Positive Meaning, with High Frequency of Both Displeased and Happy Emojis*


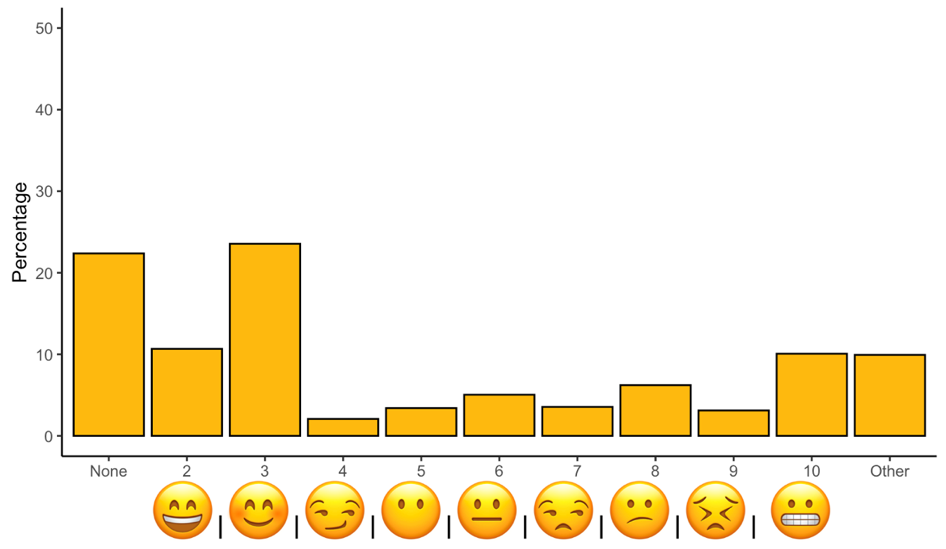


For 3 items intended to elicit negative responses and 4 items intended to elicit positive responses, responses were distributed bimodally; participants favored the “no emoji” option and emojis 6 through 10 like the negative vignettes, but also often responded with emojis 2 and 3, the happiest options. Because these loading were significant, all 7 of these items were included in future analyses, but separated into a third “ambiguous” factor to delineate the differences in loadings.

*No Significant Loadings: Vignettes Intended to Convey Either Negative or Positive Meaning with High Frequency of One Emoji Option*


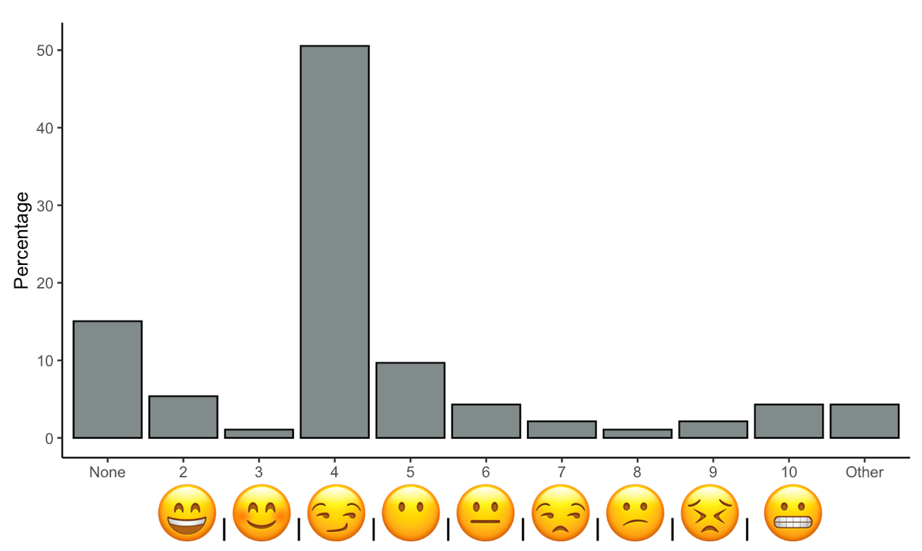


For 1 item did not load significantly onto any category (“I heard about last night”; the day after attending a party, your friend messages you. Convey to your friend that something interesting happened at the party), approximately 50% of responses were emoji 4, higher than any other item. Because this item did not load similarly to any other pattern, we excluded it from future analyses.

*No Significant Negative Loading: Vignettes Intended to Convey Negative Meaning, with Moderate Frequency of Unhappy and Neutral Emojis and Very Low Frequency of Happy Emojis*


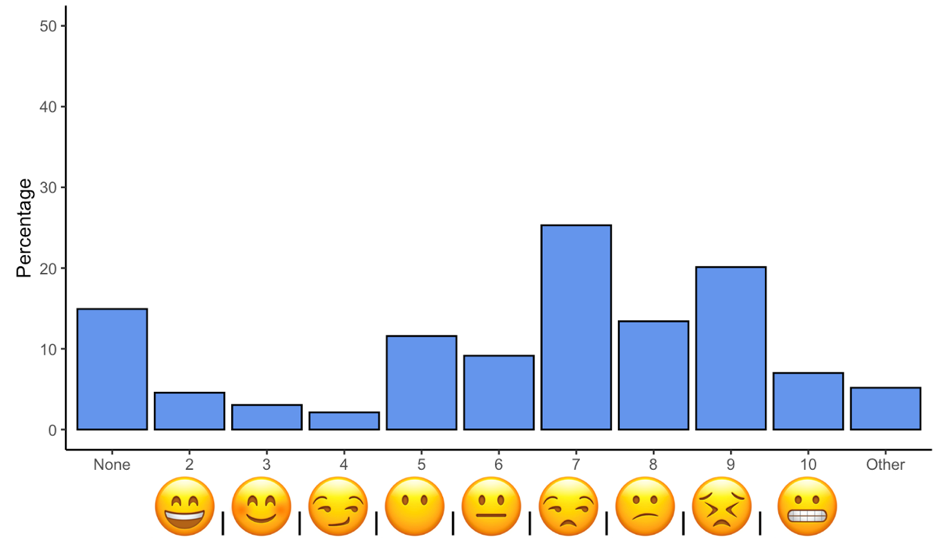


For 4 items intended to elicit negative responses but did not have a significant loading onto the negative factor, responses included a higher frequency of middle (neither very happy nor very displeased) emojis compared to other negative vignettes. These items also had fewer "no emoji" responses compared to other vignettes intended to prompt negative responses. Because they did not load similarly to the other negative vignettes, we excluded these four items from future analyses.
