# Supplementary material for: Social anxiety and emoji use: gender differences and the role of loneliness in digital communication among college students
Source: Front Psychol. 2025 Oct 23;16:1626509. doi: 10.3389/fpsyg.2025.1626509 (PMC12588911; doi:10.3389/fpsyg.2025.1626509)
Supplement: Supplementary file 7 [file Table_7.docx]

**S7 Table: Simple Slopes Results**

*Total Emoji Use (Female Only)*

| **Social Anxiety Level** | **Loneliness Test Estimate** | | **Std. Error** | **t Value** | **Pr(>\|t\|)** |
| --- | --- | --- | --- | --- | --- |
| -22.961 | -0.936 | 0.308 | | -3.036 | 0.003** |
| 2.244 | -0.452 | 0.215 | | -2.103 | 0.038* |
| 27.448 | 0.031 | 0.292 | | 0.108 | 0.915 |

*Emoji Use in Positive Contexts (Female Only)*

| **Social Anxiety Level** | **Loneliness Test Estimate** | | **Std. Error** | **t Value** | **Pr(>\|t\|)** |
| --- | --- | --- | --- | --- | --- |
| -22.961 | -1.494 | 0.325 | | -4.597 | 0.000*** |
| 2.244 | -0.914 | 0.227 | | -4.031 | 0.000*** |
| 27.448 | -0.334 | 0.308 | | -1.083 | 0.281 |

*Emoji Use in Very Negative Contexts (Female Only)*

| **Social Anxiety Level** | **Loneliness Test Estimate** | | **Std. Error** | **t Value** | **Pr(>\|t\|)** |
| --- | --- | --- | --- | --- | --- |
| -22.961 | -1.330 | 0.497 | | -2.679 | 0.009** |
| 2.244 | -0.702 | 0.346 | | -2.137 | 0.035* |
| 27.448 | -0.150 | 0.471 | | -0.319 | 0.750 |
